# Discovery of potential WEE1 inhibitors via hybrid virtual screening

**DOI:** 10.3389/fphar.2023.1298245

**Published:** 2023-12-07

**Authors:** Tingting Jin, Wei Xu, Roufen Chen, Liteng Shen, Jian Gao, Lei Xu, Xinglong Chi, Nengming Lin, Lixin Zhou, Zheyuan Shen, Bo Zhang

**Affiliations:** ^1^ Key Laboratory of Clinical Cancer Pharmacology and Toxicology Research of Zhejiang Province, Department of Clinical Pharmacology, Affiliated Hangzhou First People’s Hospital, Zhejiang University School of Medicine, Hangzhou, China; ^2^ College of Pharmaceutical Sciences, Hangzhou Institute of Innovative Medicine, Institute of Drug Discovery and Design, Zhejiang University, Hangzhou, China; ^3^ School of Electrical and Information Engineering, Institute of Bioinformatics and Medical Engineering, Jiangsu University of Technology, Changzhou, China; ^4^ Key Laboratory of Neuropsychiatric Drug Research of Zhejiang Province, School of Pharmacy, Hangzhou Medical College, Hangzhou, China; ^5^ Department of Hepatopancreatobiliary Surgery, Affiliated Hangzhou First People’s Hospital, Zhejiang University School of Medicine, Hangzhou, China

**Keywords:** virtual screening, WEE1, molecular dynamics, geometric deep learning, cell cycle

## Abstract

G_2_/M cell cycle checkpoint protein WEE1 kinase is a promising target for inhibiting tumor growth. Although various WEE1 inhibitors have entered clinical investigations, their therapeutic efficacy and safety profile remain unsatisfactory. In this study, we employed a comprehensive virtual screening workflow, which included Schrödinger-Glide molecular docking at different precision levels, as well as the utilization of tools such as MM/GBSA and Deepdock to predict the binding affinity between targets and ligands, in order to identify potential WEE1 inhibitors. Out of ten molecules screened, 50% of these molecules exhibited strong inhibitory activity against WEE1. Among them, compounds 4 and 5 showed excellent inhibitory activity with IC_50_ values of 1.069 and 3.77 nM respectively, which was comparable to AZD1775. Further investigations revealed that compound 4 displayed significant anti-proliferative effects in A549, PC9, and HuH-7 cells and could also induce apoptosis and G1 phase arrest in PC9 cells. Additionally, molecular dynamics simulations unveiled the binding details of compound 4 with WEE1, notably the crucial hydrogen bond interactions formed with Cys379. In summary, this comprehensive virtual screening workflow, combined with *in vitro* testing and computational modeling, holds significant importance in the development of promising WEE1 inhibitors.

## 1 Introduction

Various external factors such as ultraviolet, ionizing radiations and cytotoxic drugs can cause DNA damage (Ou and Schumacher, 2018). Normally, tumor cells halt cell cycle to provide time for DNA repair. WEE1 is a serine/threonine kinase and serves as a crucial regulatory protein for the G_2_/M checkpoint (Do et al., 2013; Matheson et al., 2016; Geenen and Schellens, 2017). Specifically, in response to DNA damage, WEE1 induces G_2_/M arrest by inhibitory phosphorylation of CDK at Tyr15 (Li et al., 2020). Previous research has demonstrated that over 50% of tumor cells exhibit p53 mutations that disable G_1_/S checkpoint, thereby rendering cells more reliant on the G_2_/M checkpoint (Dillon et al., 2014). Inhibition of WEE1 results in the elimination of G_2_/M arrest, allowing tumor cells to enter the division phase with incompletely-repaired DNA, finally leading to mitotic catastrophe and cell death (Vakili-Samiani et al., 2022). Moreover, WEE1 is associated with cancer progression and highly expressed in breast cancer (Ha et al., 2020), lung cancer (Liu et al., 2019), head and neck cancer (Diab et al., 2019), and ovarian cancer (Zhang et al., 2017). Consequently, WEE1 emerges as a promising target for therapeutic interventions directed at malignancies. WEE1 inhibitors can be utilized as single agents or in combination with DNA-damaging agents to enhance their therapeutic efficacy.

**FIGURE 1:**
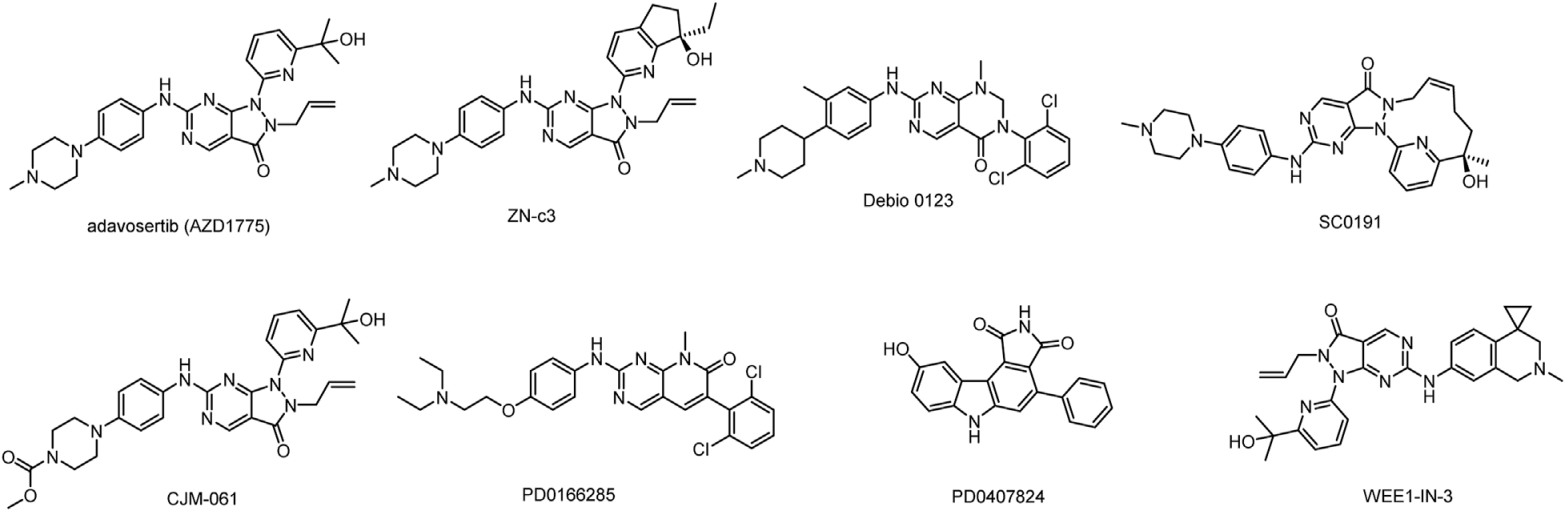
Structures of WEE1 inhibitors.

Although several WEE1 inhibitors such as Adavosertib (AZD1775), ZN-c3, IMP7-68, IMP7068, SY-4835, and Debio-0123 (Fu et al., 2018; Huang et al., 2021; Lin et al., 2022; Papadopoulos et al., 2022) have entered clinical research (Figure 1), none of them have been approved for marketing. Among these candidates, AZD1775 and ZN-c3 have achieved notable progress and are currently undergoing phase II clinical trials. Most WEE1 inhibitors have terminated clinical research due to inadequate efficacy and safety issues. Notably, AZD1775, especially in combination regimens, was associated with a heightened incidence of adverse events such as anemia, diarrhea and vomiting (Leijen et al., 2016). In contrast, ZN-c3 has exhibited superior kinase selectivity and favorable *in vivo* oral exposure and is presently in phase II clinical investigation. Therefore, the development of highly effective small molecule inhibitors possesses promising clinical prospects.

**FIGURE 2:**
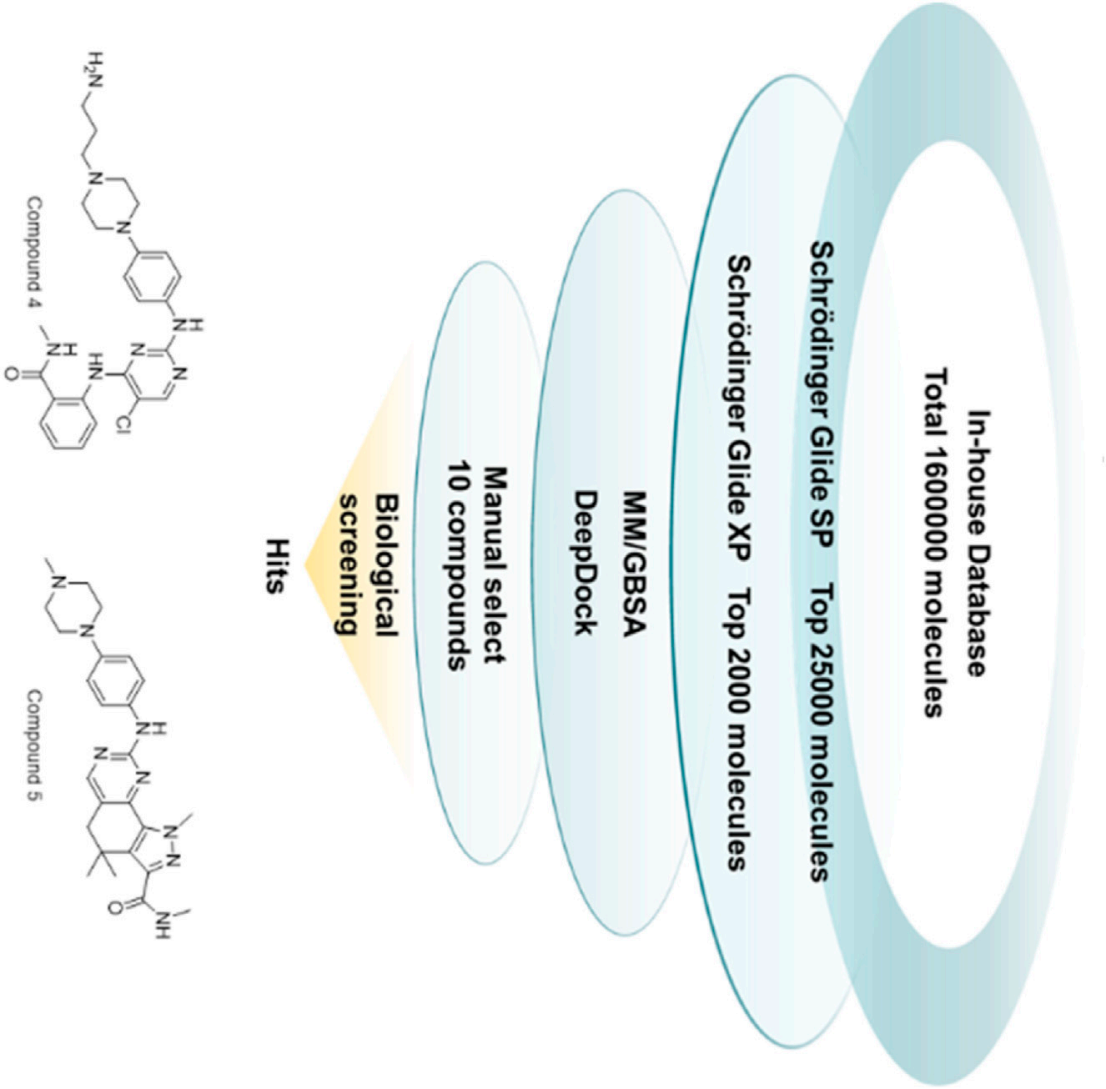
The workflow of virtual screening.

In this study, a comprehensive virtual screening workflow has designed to discover new WEE1 inhibitors with novel skeletons (Figure 2). The workflow included several processes such as Schrödinger-Glide molecular docking at different precisions, MM/GBSA, and Deepdock for high-precision evaluation. After multiple iterations, molecules with potential were screened for further evaluation. Ten molecules were screened in this study, and five of them exhibited good inhibition rates against WEE1 kinases. Compound **4** (GSK3182571) and compound **5** (Milciclib) showed comparable inhibitory activity to the positive molecules AZD1775. To explore the therapeutic potential of two compounds on tumor cells, we conducted anti-proliferation inhibitory assays in human lung cancer cells A549 and PC9, and human liver cancer cells Bel-7402 and HuH-7. Compound 4 demonstrated significant inhibitory effects on Bel-7402, HuH-7, and PC9 cells. Furthermore, we performed an analysis of apoptosis and cell cycle in PC9 cells after treated with compound 4, revealing its ability to induce apoptosis and arrest cell cycle in G1 phase. These results indicate that compound 4 has the potential to serve as a lead compound targeting WEE1, with prospects for further optimization. To gain a better understanding of the molecular interactions between compounds and their targets, we conducted molecular dynamics simulations. The investigation into the key contributions of residues around the binding pocket of compound **4** in its interaction with WEE1 could serve as a valuable theoretical reference for future structural modifications.

## 2 Result and discussion

### 2.1 Virtual screening

#### 2.1.1 Verification of docking parameters

In the present study, we investigated the protein structures with the aim to discern strategies that could enrich high-affinity compounds specific to WEE1. High-definition structural data for these proteins were sourced from the RCSB Protein Data Bank. Specifically, the human-originated WEE1 protein structure, labeled 5VD8, was assessed, boasting a resolution of less than 2Å.

**FIGURE 3:**
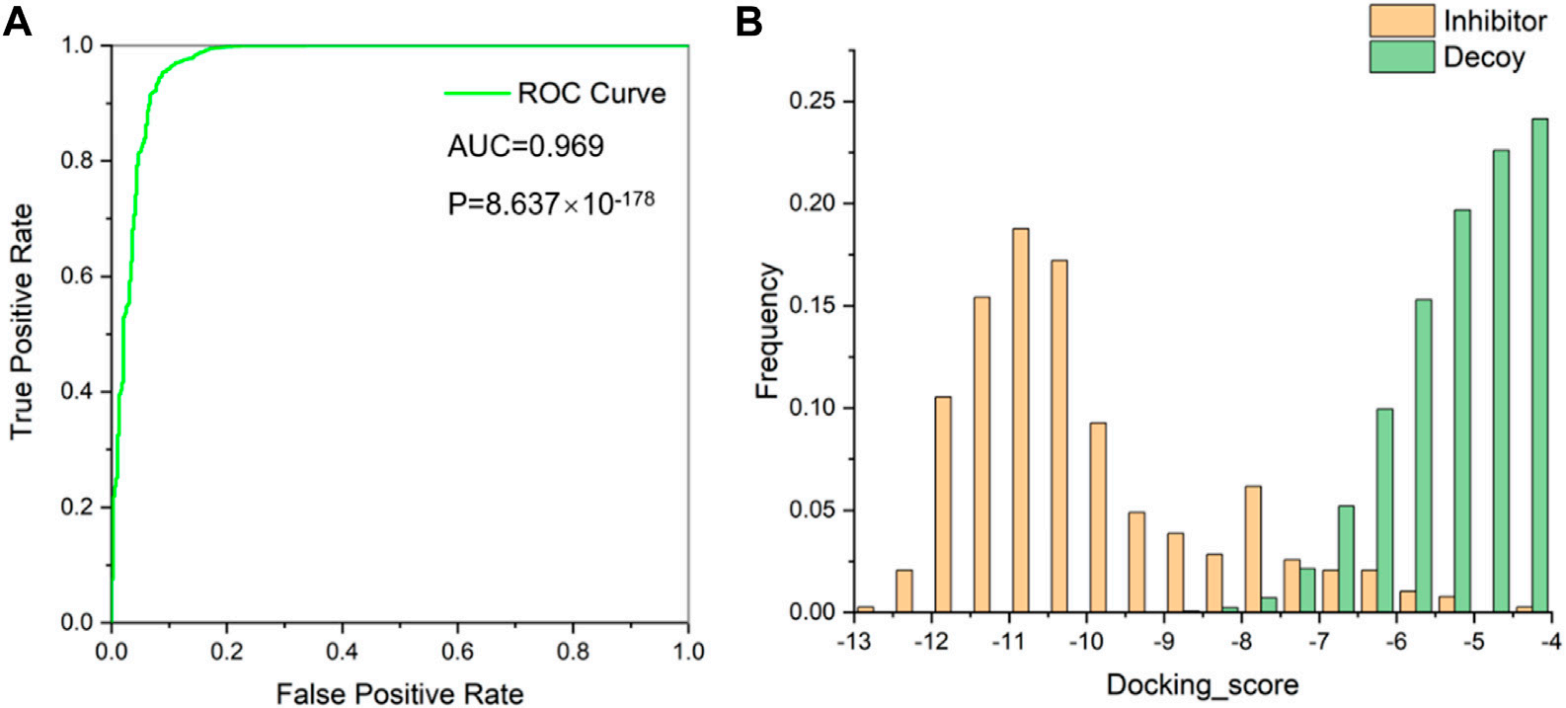
**(A)** Receiver operating characteristic (ROC) curve for evaluating virtual screening methods; **(B)** Docking score distribution of WEE1 inhibitors and decoys.

This protein was subjected to further preparation via the Schrödinger’s Protein Preparation Wizard module. Subsequent evaluations of the prepared proteins were conducted using the Glide_SP to ascertain the efficacy of these techniques and protein chains in differentiating between inhibitors and decoys. A substantial dataset, consisting of 389 validated WEE1 inhibitors obtained from ChEMBL database (Stein et al., 2021) and 139,320 random decoys acquired through DUDE-Z method (Mendez et al., 2019), was created, and these ligands were docked to the protein pocket. Throughout this docking procedure, scores were documented, facilitating the calculation of *p*-values. The achieved area under the curve (AUC) was 0.969 with a *p*-value falling below 0.001 (Figure 3A). This remarkable statistical significance underscores the robustness of our method in distinguishing between inhibitors and decoys. A frequency histogram evaluation illuminated that 5VD8 exhibits exemplary discriminatory capabilities and was thus selected for further screening processes (Figure 3B).

#### 2.1.2 Preparation “in-house” database and WEE1 protein

Our “in-house” database currently contains 160,0000 unique molecules sourced from the Maybridge compound database and MCE drug-like compound database. To ensure data accuracy, we utilized RDKit to preprocess and eliminate any redundant molecular structures. The resulting molecules were then transformed into 3D structures using Schrödingers LigPrep module.

The protein structure with PDB ID 5VD8 was retrieved from the RCSB protein database (https://www.rcsb.org/) for further preparation. The Protein Preparation Wizard in Schrödinger 2021–2 suite was used for protein preparation, which involved filling in missing hydrogens, partial charges, side chains, and loops, optimizing hydrogen bond assignments, and minimizing energy.

#### 2.1.3 Comprehensive virtual screening workflow

A multi-stage process was designed, which consisted of several key components. The initial screening was performed by Schrödinger-Glide molecular docking, using varying levels of precision to identify promising candidates. The selected molecules were then further evaluated and refined by MM/GBSA and Deepdock. This iterative and comprehensive screening approach ensures efficient exploration of potential WEE1 inhibitors. In this study, the Glide docking tool in the Schrödinger package was utilized for screening molecules in the internal database. The screening process consisted of two rounds, with the first round using standard precision (SP) to select the top 25,000 molecules with a score lower than or equal to −6.5 kcal/mol. The second round used ultra-precision (XP) with stricter restrictions on ligand-receptor shape complementarity, resulting in the selection of 2000 molecules with a score lower than or equal to −8 kcal/mol (Vass et al., 2012). MM/GBSA calculation and the geometric deep learning framework algorithm Deepdock were then used to predict the molecular and target affinity of the selected molecules (Sándor et al., 2010). Compared to traditional docking scoring functions, MM/GBSA has higher accuracy in predicting binding affinity by taking into account both molecular mechanical energy and solvation energy. The study followed standard MM/GBSA procedures as outlined in Schrödinger, including the preparation of the protein-ligand complex and energy minimization using the OPLS4 force field (Jorgensen et al., 1996; Halgren et al., 2004; Friesner et al., 2006). The use of the Deepdock model further improved the understanding of the interaction between small molecules and WEE1 protein by predicting the binding mode between ligand and target protein.

**FIGURE 4:**
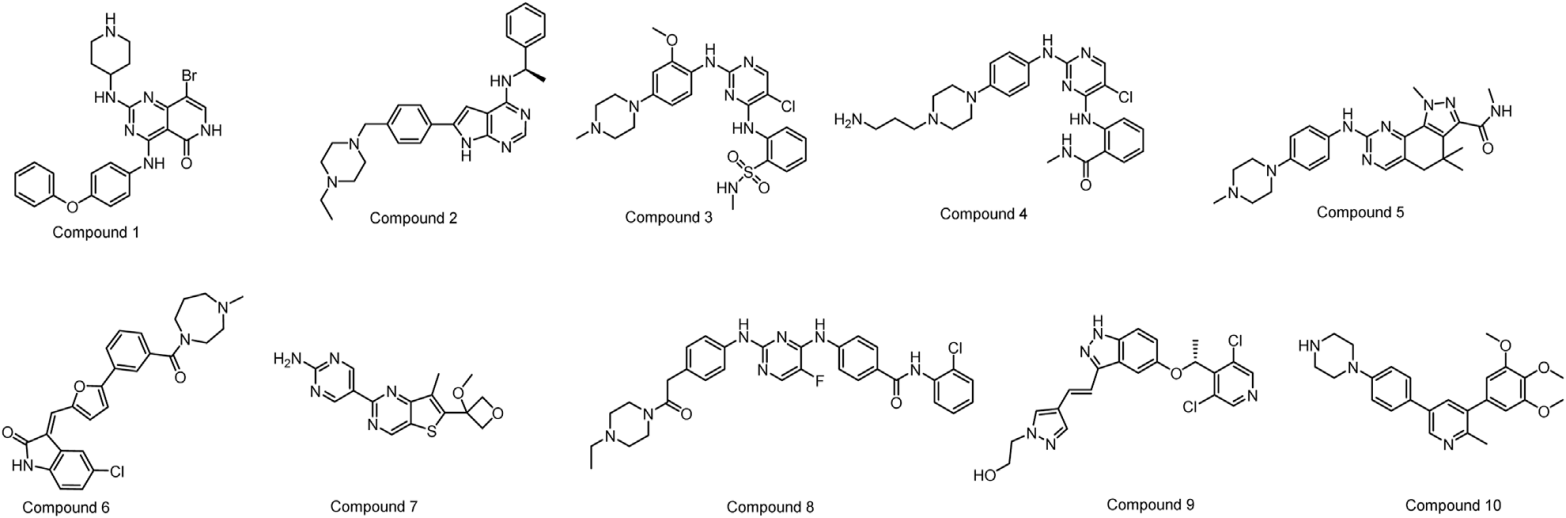
The structures of the ten selected compounds.

From the remaining molecules, a manual selection process was used to identify 10 compounds (Figure 4; Table 1). This selection method took into account the interaction pattern scores and molecular combinations from different software, while also excluding molecules with incorrect binding patterns. We then conducted empirical assessments by evaluating the binding scores, binding modes (with a focus on critical amino acid interactions), inherent properties of the molecules (molecular weight less than 600, hydrogen bond donor number less than 5, hydrogen bond acceptor less than 10, topologically polarized surface (TPSA) less than 120, rotational bond number (RotB) less than 10, calculated value of lipid water partition coefficient (clogP) less than 5, etc.), and molecular synthesizability. This determination aimed to identify molecules with higher binding affinities, while excluding those lacking potential for drug development.

**TABLE 1:** The docking score and inhibition rate of the selected 10 small molecules.

Entry	Docking score (SP)(kcal/mol)	Docking score (XP)(kcal/mol)	MM-GBSA ΔG Bind(kcal/mol)	Deep dock score	Inhibition Rate(%)
**Compound 1**	−9.205	−9.018	−76.921	−121.682	64.18 ± 1.16
**Compound 2**	−8.566	−9.207	−71.743	−166.292	134.70 ± 1.61
**Compound 3**	−7.825	−8.778	−70.275	−266.036	127.17 ± 1.08
**Compound 4**	−9.075	−10.01	−71.062	−256.034	126.59 ± 1.66
**Compound 5**	−9.612	−11.083	−75.997	−231.753	137.84 ± 1.08
**Compound 6**	−8.678	−8.57	−81.389	−158.969	24.75 ± 2.33
**Compound 7**	−8.632	−7.191	−81.189	−138.888	11.18 ± 0.04
**Compound 8**	−9.129	−6.280	−75.282	−247.979	95.45 ± 2.24
**Compound 9**	−9.476	−10.367	−79.068	−173.712	58.18 ± 4.03
**Compound 10**	−9.393	−9.781	−80.855	−91.280	32.77 ± 0.22

### 2.2 Biological evaluation

#### 2.2.1 Inhibitory activity assay

**FIGURE 5:**
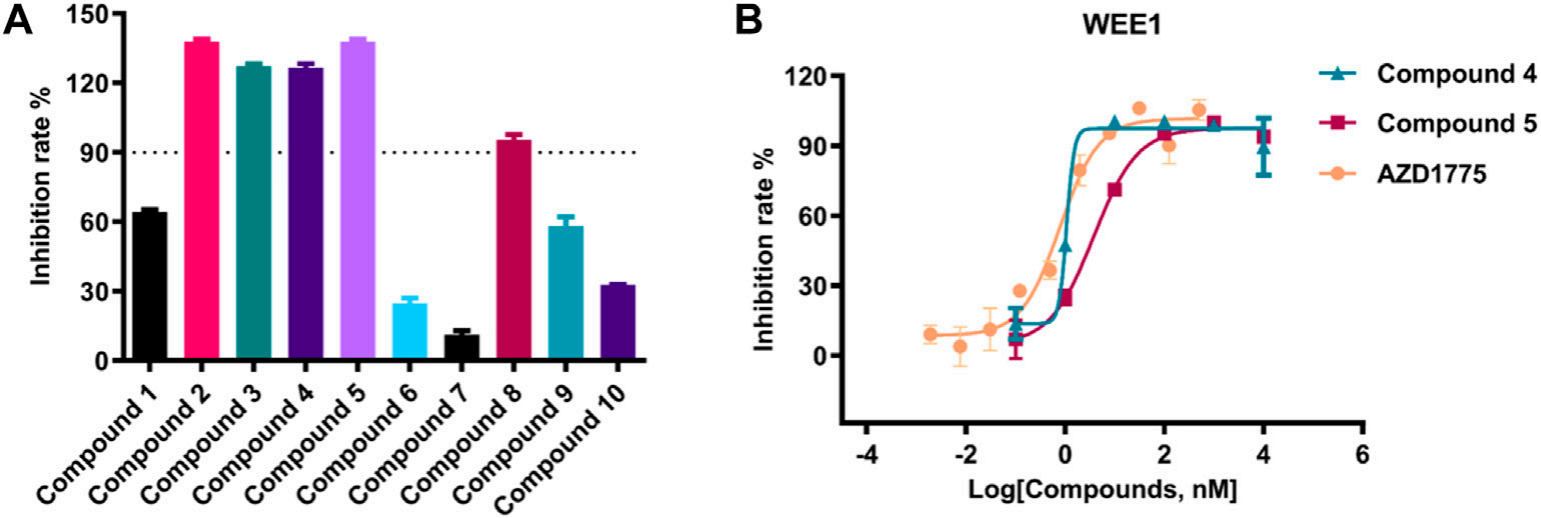
**(A)** The inhibition rate (%) assay of ten selected compounds (25 μM) against WEE1; **(B)** The IC_50_ value of compound **4**, compound **5** and AZD1775 against WEE1 kinase.

The kinase activity of these ten compounds obtained by virtual screening were evaluated. Compounds 2, 3, 4, 5, and 8 exhibited a strong inhibitory effect on WEE1 protein, with an inhibition rate of almost 100% at a concentration of 25 μM (Figure 5; Table 2). Consequently, these five molecules were selected for further testing to determine their half maximal inhibitory concentration (IC_50_). The results indicated that five compounds displayed good inhibitory activity, with IC_50_ values less than 1 μM. Compound **4** and Compound **5** showed strongest inhibitory effect, displaying IC_50_ values of 1.069 and 3.77 nM, respectively. These IC_50_ values were comparable to the activity result of the positive control AZD1775, with an IC_50_ value of 0.786 nM. Therefore, the anti-proliferative activity of these two compounds was investigated in further studies.

**TABLE 2:** The IC_50_ value of five selected compounds against WEE1 kinase.

	Compound 2	Compound 3	Compound 4	Compound 5	Compound 8	AZD1775
WEE1 IC_50_ (nM)	102.7	988.4	1.069	3.77	796.9	0.786

#### 2.2.2 *In vitro* antiproliferative activity

**FIGURE 6:**
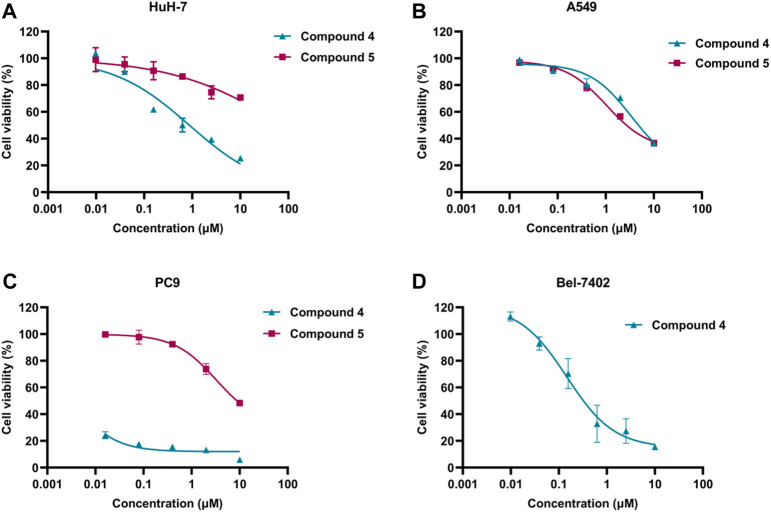
**(A)** The IC_50_ value of compound **4** and compound **5** against HuH-7 cell; **(B)** The IC_50_ value of compound **4** and compound **5** against A549 cells; **(C)** The IC_50_ value of compound **4** and compound **5** against PC9 cells; **(D)** The IC_50_ value of compound **4** against Bel-7402 cells.

As shown in Figure 6, The anti-proliferation activity of compound **4** and compound **5** against lung cancer (A549 and PC9) and liver cancer (HuH-7) cells were studied. The results indicated that compound **4** exhibited better anti-proliferation activity than compound **5** in 3 cells. Moreover, PC9 cells demonstrated higher sensitivity to compound **4**, with an IC_50_ value of 0.44 nM. Meanwhile, compound **4** displayed anti-proliferation activity against A549 and HuH-7 cells with the IC_50_ values 5.171 and 0.875 μM, respectively. Additionally, compound **4** was assessed for its anti-proliferation activity on liver cancer cell Bel-7402, and it exhibited promising activity with an IC_50_ of 0.147 μM. Thus, the kinase and cell antiproliferative activity evaluation suggested that compound **4** could be a potential WEE1 inhibitor, and therefore, it may be selected as the lead compound for structural optimization.

#### 2.2.3 Cell apoptosis analysis

**FIGURE 7:**
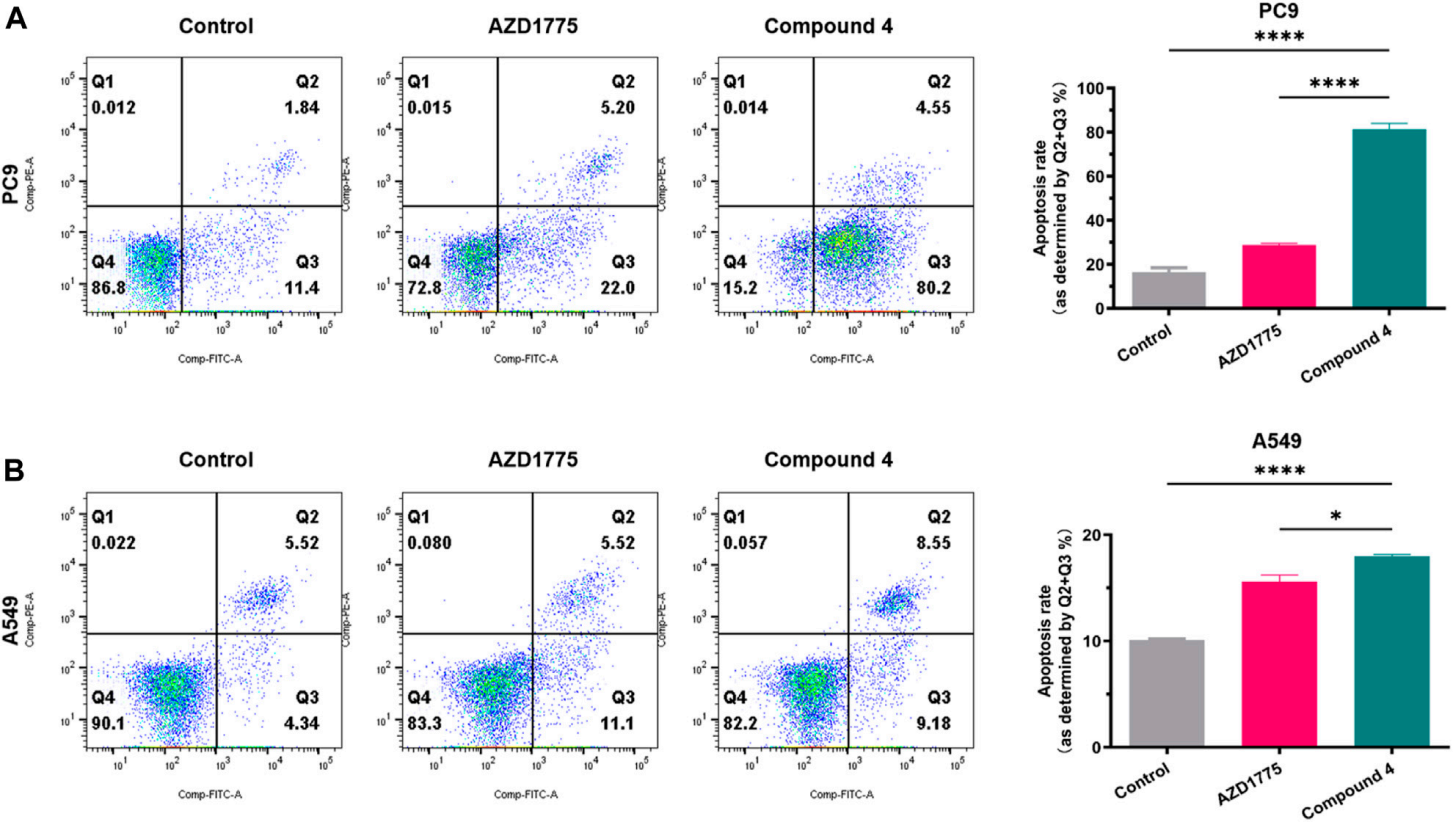
Cell apoptosis analysis of compound 4. **(A)** PC9 cells were treated with AZD1775 or compound **4** (500 nM) for 72 h; **(B)** A549 cells were treated with AZD1775 or compound **4** (2 μM) for 72 h. Apoptosis rate = Q2% (late apoptosis rate) + Q3% (early apoptosis rate), (ANOVA multiple comparison tests).

To validate the inhibitory effects of WEE1 inhibitors in PC9 and A549 cells, Annexin V/PI was utilized to stain apoptotic cells (early apoptosis + late apoptosis). After PC9 cells were treated with 500 nM compound 4 or AZD1775 for 72 h, the percentage of apoptotic cells reached to 13.24%, 27.20%, and 84.75% in blank control group, AZD1775 group and compound **4** group, respectively. A549 cells were treated with 2 μM compound 4 or AZD1775 for 72 h, the percentage of apoptotic cells reached to 9.86%, 16.62%, and 17.73% in blank control group, AZD1775 group and compound **4** group, respectively. These results suggest that compound 4 is more effective than AZD1775 in inducing apoptosis in PC9 cells. Furthermore, compared to A549 cells (p53 wild type), compound 4 or AZD1775 can induce more apoptosis in PC9 cells (p53 mutant) (Figure 7).

#### 2.2.4 Cell cycle analysis

**FIGURE 8:**
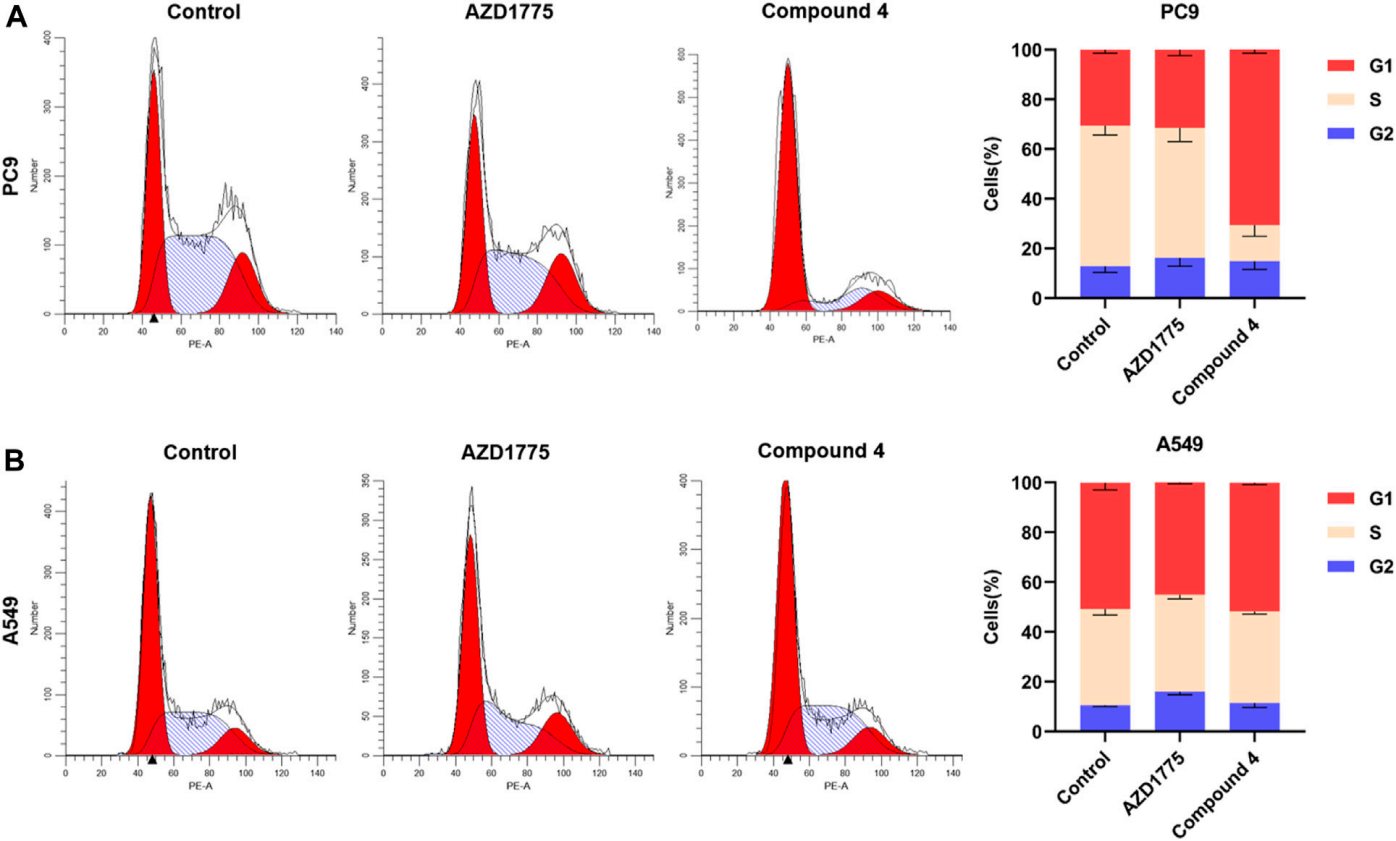
Cell cycle analysis of compound 4. **(A)** PC9 cells were treated with AZD1775 or compound **4** (500 nM) for 24 h; **(B)** A549 cells were treated with AZD1775 or compound **4** (2 μM) for 24 h.

As shown in Figure 8, the impact of compound **4** on cell cycle distribution of PC9 and A549 cells were studied by flow cytometry using propidium iodine (PI) staining. Results showed that when PC9 cells were treated with 500 nM compound **4** for 24 h, the proportion of cells in the G_1_ phase was significantly increased compared to the blank group, while the proportion of cells in the G_2_ phase did not change significantly. However, after A549 cells were treated with 2 μM compound 4 or AZD1775 for 24 h, there was no significant change on cell cycle distribution. These findings suggested that WEE1 inhibitor abolished G_2_/M phase arrest and increased the proportion of G_1_ phase cells, likely due to compound **4** also appeared to exert significant effects on the G_1_/S checkpoint. Moreover, in comparison to cells with wild-type p53, WEE1 inhibitors exert a more pronounced impact on the cell cycle of cells with p53 mutations.

### 2.3 Molecular dynamics and binding mode analysis

**FIGURE 9:**
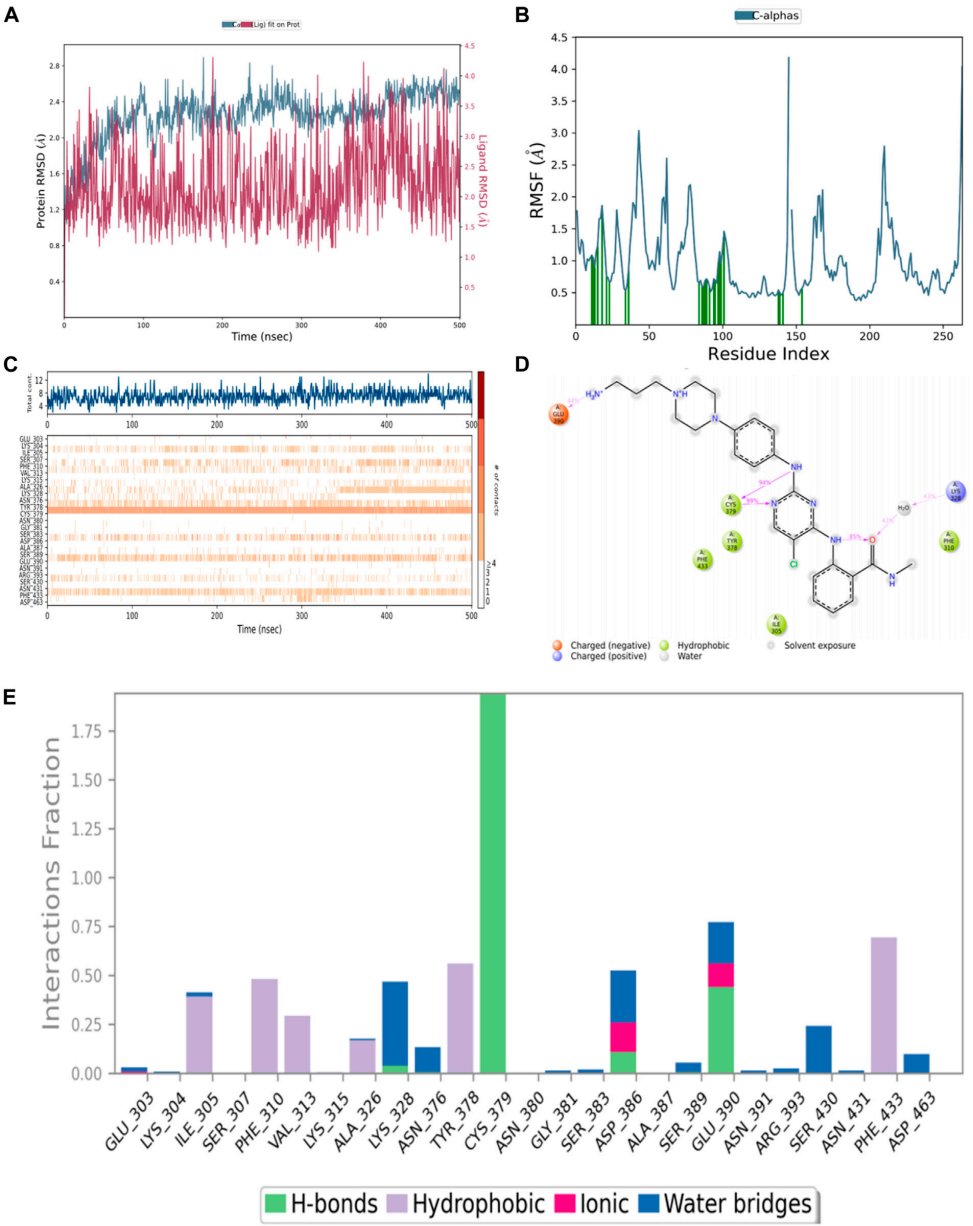
**(A)** RMSD value of compound **4** (red) and WEE1 (blue); **(B)** The RMSF profile of WEE1 (PDB ID 5VD8) which represents local changes along the protein chain throughout simulation trajectory; **(C)** Timeline representation of WEE1-compound **4** interactions; **(D)** The 2D diagram of protein-ligand interaction. The purple arrows represented H-bond interactions and water bridges; **(E)** The major contributions of individual amino acid residues to the compound 4-WEE1 complex.

According to the results of biological evaluation, compound **4** was selected for MD simulation. The MD simulation was performed for 500 ns, and equilibrium was reached at 100 ns (Figure 9A). Figure 9B shows the RMSF plot for WEE1, indicating that the fluctuation of the amino acid residue index ranges from 0.5 Å to 4.5 Å. As demonstrated in Figure 9C, the data indicate that Cys379 interacted with compound 4 at nearly all time points. To explain these observations, we selected the last conformation for further analysis and plotted a schematic illustrating the detailed ligand atom interactions with specific protein residues. We observed that the hydrogen bond interactions with Cys379 mainly involved the nitrogen and amino groups on the pyrimidine of compound **4**, as indicated in Figures 9D,E. In addition, compound **4** also interacts with Glu390 of WEE1 protein through hydrogen bonding interactions, which is different from that of AZD1775. Compound **4** interacts with Lys328 through water bridge interactions. The amino group on the benzene ring creates an intramolecular hydrogen bond with the carbonyl group, serving to stabilize the compound’s conformation. Importantly, this interaction of Cys379 is a well-established model and has also been observed in other WEE1 inhibitors.

## 3 Conclusion

This research has utilized a robust virtual screening workflow to identify potential WEE1 inhibitors. Compounds **4** and **5** have been identified as potent inhibitors of WEE1 kinases. Particularly, compound **4** has demonstrated significant therapeutic potential against various cancer types, exhibiting inhibitory and anti-proliferation characteristics that are comparable to, if not better than, existing WEE1 inhibitors. The investigation of compound **4**′s ability to induce apoptosis and its impact on the cell cycle further highlights its potential application in cancer treatment. These results confirm that compound **4** is a novel WEE1 inhibitor, which can be used as a potential lead compound for the further development of highly active molecules targeting the WEE1 signaling pathway. However, our study still has some limitations. For instance, the selectivity of compound 4 towards other kinases, as well as the pharmacokinetic properties and safety of compound 4, necessitate further experimental validation. In summary, these findings contribute significantly to the scientific understanding of WEE1 inhibitors and provide a foundation for future refinement of molecular structures and laboratory optimization.

## 4 Material and methods

### 4.1 Compounds

Compound 1 (Denfivontinib, HY-12333), Compound 2 (AEE788, HY-10045), Compound 3 (ALK inhibitor 2, HY-15358), Compound 4 (GSK3182571, HY-12400), Compound 5 (Milciclib, HY-10424), Compound 6 (CX-6258 hydrochloride, HY-18095B), Compound 7 (GNE-317, HY-12763), Compound 8 (TCS7010, HY-70061), Compound 9 (LY2874455, HY-13304) and Compound 10 (LDN-214117, HY-16712) were purchased from MedChemExpress (Junction, NJ, United States).

### 4.2 Protein and ligand preparation

The X-ray co-crystallized protein structure (PDB ID: 5VD8) was obtained from the RCSB Protein Database for further protein preparation (Zhu et al., 2017; Burley et al., 2023). Among the total of WEE1 protein structures recorded in the RCSB database, PDB ID: 5VD8 was selected as our target for virtual screening after filtering based on species and non-covalent ligand. The protein preparation process, including preprocessing, hydrogen bond assignment, and restrained minimization, was carried out using the Protein Preparation Wizard module from the Schrödinger 2021–2 suite (Sastry et al., 2013; Schrödinger, 2021a). During the preprocessing step, any issues with the protein were identified and resolved using default parameters, such as filling in missing side chains and loops. The hydrogen bond assignments were then optimized by sampling water orientations and using PROPKA at a pH of 7.4 (Rostkowski et al., 2011). Finally, a restrained energy minimization was performed, using the OPLS4 force field to converge the heavy atoms to an RMSD of 0.30 Å (Lu et al., 2021).

### 4.3 Database preparation

The in-house database used for screening was comprised of two components, the commercial Maybridge compound database and MCE’s drug-like molecule library. The preparation of all ligands was carried out using the default parameters of the LigPrep module from the Schrödinger 2021–2 suite (Schrödinger, 2021b). The Epik (version 5.6) program was utilized to perform hydrogenation, salt removal, tautomer generation, and calculation of ionization states, all under the OPLS4 force field and at a pH of 7.0 ± 2.0 (Shelley et al., 2007; Greenwood et al., 2010; Schrödinger, 2021c). Additionally, up to 32 stereoisomers can be generated for each ligand while preserving the specific chirality under the computational conditions.

### 4.4 Receptor grid generation

To prepare for the subsequent docking stage, a file was generated containing the center coordinates of the protein pockets. The central coordinates of the original ligand were selected as a reference for the docking process. Subsequently, the original ligands were deleted, resulting in the creation of the grid file. These steps were performed using the receptor mesh generation module from the Schrödinger 2021–2 suite.

### 4.5 Docking based virtual screening

The virtual screening workflow described in this paper consists of a series of filtering steps, each increasing in precision. The process began by selecting the top 250,000 molecules from the in-house database through the use of the SP precision in Glide docking. These molecules were then screened through the XP precision of Glide docking, where 10 docking conformations were established for each compound. In the third step, 20,000 molecules obtained from the previous round of Glide docking were filtered through MM/GBSA to identify the molecules with the highest scores, which were determined using a combination of DeepDock and manual selection. Finally, 10 molecules were chosen for further analysis of their bioactivity.

#### 4.5.1 Glide docking

The Glide molecular docking module of Schrödinger’s suite is a widely-used tool in the field of computational drug discovery, allowing for the prediction of the 3-dimensional structure of a small molecule when in complex with a protein target (Friesner et al., 2004). These interactions, determined by physical and chemical properties such as shape complementarity, hydrogen bonding, and electrostatic interactions, are crucial for understanding the efficacy and specificity of a drug candidate.

In this study, both the Glide SP and XP precision were utilized. The receptor grid file of the WEE1 target and ligand file were loaded into the system and the OPLS4 force field was selected. The desired precision level was set to determine the accuracy of the simulation, and the maximum number of output structures and number of poses per ligand were set to 5. The docking simulation was initiated by running the program.

#### 4.5.2 MM/GBSA

The MM/GBSA method, which stands for Molecular Mechanics Generalized Born Surface Area, is a widely used computational technique for predicting the binding affinity of a small molecule to a protein target in computational drug discovery (Rastelli et al., 2010; Genheden and Ryde, 2015). It integrates the benefits of molecular mechanics and generalized Born methods to provide a precise and efficient estimation of binding free energy. In this study, the binding free energy was calculated using the MM/GBSA calculation method. The WEE1-ligand complexes obtained from docking were optimized using the local optimization feature in Prime wizard of Maestro (Schrödinger Release 2021–2) (Jacobson et al., 2002; Jacobson et al., 2004; Schrödinger, 2021d). The force field employed in this calculation was OPLS4, and the binding energy was determined based on a set of receptor and ligand. The equation used to calculate the binding free energy was as follows:

ΔG (bind) = ΔG (solv) + ΔE (MM) + ΔG (SA). The binding free energy was calculated using the MM/GBSA method, which combines molecular mechanics and generalized Born methods. The minimized energy of the protein-ligand complexes, represented as ΔE (MM), was determined using the OPLS4 force field. The solvation energy variance between the protein-ligand complexes and the sum of the solvation energies for the protein and ligand, represented as ΔG (Solv), was also considered. Furthermore, the difference in surface area energies for the complexes, represented as ΔG (SA), was included in the calculation. The minimization of the docked complexes was conducted using the local optimization feature of Prime in Maestro (Schrödinger Release 2021–2).

#### 4.5.3 Deepdock

The DeepDock program on GitHub is a deep learning framework designed for protein-ligand docking, a computational approach that predicts the binding affinity between a protein and a small molecule (Liao et al., 2019). This program uses a convolutional neural network (CNN) to learn the correlation between the three-dimensional structures of the protein and the ligand and their binding affinity. The process started with the application of the masif algorithm to determine the interfacial characteristics of WEE1 when bound to a molecule.

The program inputs 2D molecular and protein pocket graphs and generates continuous representations. Based on these representations, it calculates a statistical potential based on the likelihood of distances between the protein and the molecule. Finally, it employs an optimization technique to produce the binding configuration of the molecule.

### 4.6 Inhibitory activity assay

The tested compounds were dissolved in DMSO to make a 10 mM stock solution, which was further diluted to a drug solution of 25 µM. Initially, 2× ATP and substrate solution and 2× kinase and metal solution were prepared using assay buffer (Hepes 50 mM, MgCl2 2 mM, Brij35 0.01%, EGTA 1 mM, DTT 2 mM and ddH2O.). Transfer 20 nL compound to 384 assay plate by Echo 655. Then, 2 μL of 2× kinase and metal solution was mixed and incubated in a 384 assay plate for 10 min at 25°C. 2 μL of 2× substrate & ATP solution were added to the well, and incubated at 25°C for 60 min 4 μL of ADP-Glo reagent was added to the well, and incubated for 40 min at 25°C. 8 μL of kinase detection reagent was added to the well, and incubated for 40 min at 25°C. Read the RLU (relative light-emitting unit) signal using the BMG multifunction microplate reader, and the signal intensity is used to characterize the degree of kinase activity. We used Staurosporine (10 µM) as a positive control compound to calculate the relative inhibition rate. Staurosporine is a prototypical ATP-competitive kinase inhibitor in that it binds to many kinases with high affinity. The inhibition rates were calculated using the equations (X: log of inhibitor concentration; Y: % Inhibition). Assays were performed on three independent experiments.

Y=Bottom+Top – Bottom/(1+10^ (LogIC50 – X×hillslope))

### 4.6 Flow apoptosis and cell cycle detection

For apoptosis detection, Cells were performed with Pharmingen FITC Annexin V Detection KitⅠ(BD Bioscience, Oxford, UK). Briefly, Cells were harvested and washed once with 1×binding buffer and then incubated with Annexin V and Propidium Iodide (PI) for 15 min in the dark at 37°C.

For cell cycle detection, Cells were harvested and gently washed with PBS, and fixed with 70% cold ethanol −20°C overnight. After centrifugation, Cells were retained for precipitation and incubated with PI/RNase Staining Buffer (BD Bioscience, Oxford, United Kingdom) for 15 min in the dark at 37°C.

All samples were acquired by flow cytometry (FACS CantoⅡ, BD Bioscience) and analysed with Flowjo software version 10.4.

### 4.7 Anti-proliferation activity assay

Anti-proliferation activity assay was detected by Cell Counting Kit-8 detection kit (CCK-8, #K1018) provided by Apexbio (United States). In brief, cells were seeded in 96-wells plates at a density of 3 × 10^3^/well for 24 h. Then, cells were treated with indicated concentrations of compounds for 72 h. Supernatant was totally removed, and 100 μL of CCK-8 solution was added to each well and cultured for another 1 h at 37°C. The absorbance of plates was measured by SpectraMax M2 (Molecular Devices, San Jose, CA, United States) at 450 nm. The inhibition rates were calculated for each wells as (1-(OD450 treated cells/OD450 control cells)) × 100%. Assays were performed on three independent experiments.

### 4.8 Molecular dynamics

The compound **4** selected for complex formation with WEE1 underwent molecular dynamics simulations. The initial structure of WEE1-compound 4 used for MD simulations was obtained by extra-precision (XP) docking (Schrödinger, 2021e). It was first processed through the Protein Preparation Wizard module. The input system was built using the System Builder module from the Schrödinger 2021–2 Suite. With default parameters, the complex molecule was placed at the center of a box filled with SPC water molecules, and the ensemble class for molecular dynamics simulations was set to NPT. The temperature was kept at 300K, and the boundaries of the box were established 10 Å away from the farthest radius of the protein. 0.15 M NaCl, was added to balance the system charge, with energy minimized to 100 ps? The prepared model contained 34,412 atoms was loaded into the Molecular Dynamics module for further simulation. The simulation duration was set at 500 ns, with a recording interval of 1 ns for each recording. The dynamics simulations were performed under OPLS4 force field at the temperature of 300 K and the pressure of 1.01325 bar. The results of the simulations were analyzed using Simulation Interaction Analysis Diagram.

### 4.9 Statistical analysis

The results were presented as mean ± SD of three independent experiments. We analyzed data with GraphPad Prism 8.0 software (San Diego, CA, United States). The significance of differences between two groups were determined by unpaired Student’s t tests and ANOVA multiple comparison tests. Statistically significant *p* values were labelled as: *p < 0.05, **p < 0.01, ***p < 0.001, ****p < 0.0001.

## Data Availability

The raw data supporting the conclusion of this article will be made available by the authors, without undue reservation.
