# Supplementary material for: Discovery of potential WEE1 inhibitors via hybrid virtual screening
Source: Front Pharmacol. 2023 Dec 7;14:1298245. doi: 10.3389/fphar.2023.1298245 (PMC10740156; doi:10.3389/fphar.2023.1298245)

Virtual screening workflow → Biological evaluation → Molecular binding mode analysis

Virtual screening workflow

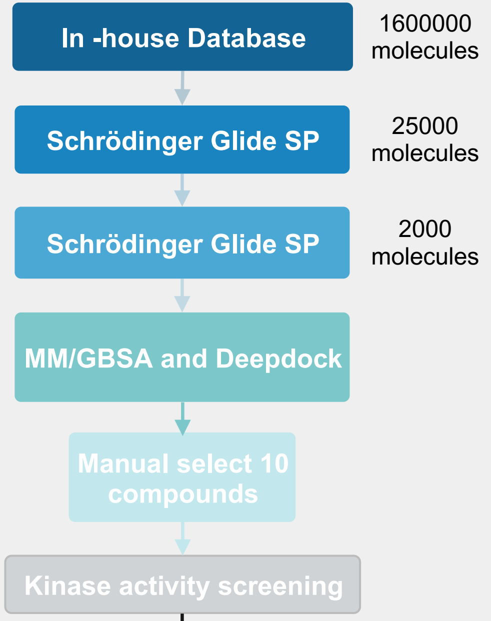

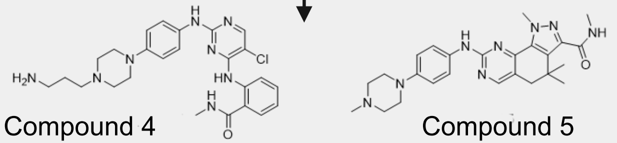

Compound 4 Compound 5

Biological evaluation

The IC_{50} value of compound 4/5 against cancer cell		
Cell line	Compound 4	Compound 5
PC9	0.44 nM	8.88 μM
HuH-7	0.88 μM	80.01 μM

Cell apoptosis analysis in PC9 cells		
Control	AZD1775	Compound 4
5.61%	14.31%	48.19%

Molecular binding mode analysis

The 2D diagram of WEE1-compound 4 interaction

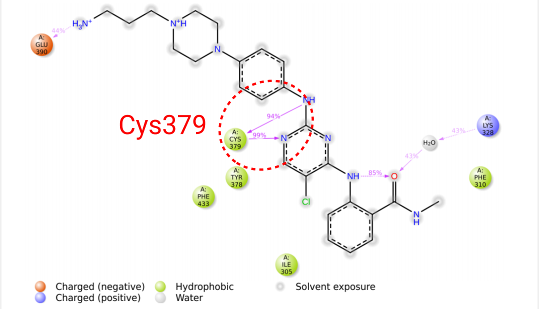

Compound 4 mainly interacts with Cys379 of WEE1 protein

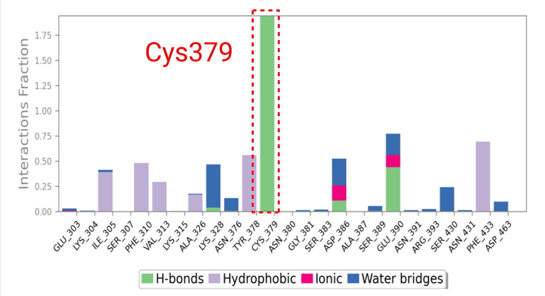

Supplement: Supplementary file 1 [file Image1.PDF]
